# A TDR-Based Soil Moisture Monitoring System with Simultaneous Measurement of Soil Temperature and Electrical Conductivity

**DOI:** 10.3390/s121013545

**Published:** 2012-10-09

**Authors:** Wojciech Skierucha, Andrzej Wilczek, Agnieszka Szypłowska, Cezary Sławiński, Krzysztof Lamorski

**Affiliations:** Institute of Agrophysics, Polish Academy of Sciences, ul. Doświadczalna 4, 20-290 Lublin, Poland; E-Mails: a.wilczek@ipan.lublin.pl (A.W.); a.szyplowska@ipan.lublin.pl (A.S.); c.slawinski@ipan.lublin.pl (C.S.); k.lamorski@ipan.lublin.pl (K.L.)

**Keywords:** soil moisture, TDR technique, monitoring stations

## Abstract

Elements of design and a field application of a TDR-based soil moisture and electrical conductivity monitoring system are described with detailed presentation of the time delay units with a resolution of 10 ps. Other issues discussed include the temperature correction of the applied time delay units, battery supply characteristics and the measurement results from one of the installed ground measurement stations in the Polesie National Park in Poland.

## 1. Introduction

The global hydrologic cycle and the functioning of ecosystems depend on the complex interactions between soil, vegetation, and the atmosphere. An increasing amount of evidence suggests that these interactions play a larger role in regulating atmospheric conditions than was initially assumed [1–3].

With the development of climate models, researchers are becoming increasingly aware of the critical role of soil water availability in simulating water fluxes over land surfaces [4]. Models that do not consider the impact of rainfall pulses and precipitation regime changes on evapotranspiration [5] and total ecosystem respiration [6] will not accurately model the accompanying climatic responses [7,8]. Spatial and temporal variations in soil moisture can have a lasting impact on climate factors such as precipitation [9], and the inclusion of sub-grid scale soil moisture heterogeneity can improve the performance of global climate models [10].

In the past, information about soil moisture was obtained by laboratory analysis of soil samples or from daily to biweekly measurements taken using *in situ* soil moisture probes. These methods have drawbacks, namely low temporal resolution and/or high labour requirements.

Time domain reflectometry (TDR) is a well-known method for measuring soil water content and electrical conductivity. Both of these quantities are important for a variety of hydrological processes and the interaction between soil and atmosphere for climate predictions. The first application of TDR to soil water measurements was reported by Topp *et al*. [11]. The main advantages of TDR over other soil water content measurement methods are: (i) superior accuracy to within 1 or 2% of volumetric water content; (ii) calibration requirements are minimal—in many cases soil-specific calibration is not needed, but soil-specific calibration is possible for the applications that demand high accuracy; (iii) lack of radiation hazard associated with the neutron probe or gamma-rays attenuation techniques; (iv) application of TDR probes can give an excellent spatial and temporal resolution; (v) measurements are rapid, non-destructive and simple to obtain, and (vi) the method is capable of providing continuous measurements through automation and multiplexing. TDR probes of custom design or purchased from numerous vendors are comprehensively described in the aspects of construction details, material specific calibration and the waveform interpretation [12,13]. There are numerous applications for determining the spatial distribution of water content in soils [14] and snow [15], for water content determination in woody biomass [16] and wood materials [17,18] or for the purpose of irrigation scheduling [19]. TDR measurement equipment was also successfully implemented in particular study cases [20,21]. TDR soil moisture meters are commercially available, however their relatively high price limits the applications of this measurement technique mainly to scientific research in hydrology [19], optimization of soil irrigation techniques [22], or the soil surface layer moisture monitoring for the purpose of validation and calibration of satellite images used for assessing the influence of the soil moisture on the climate on global scale [23].

The objective of this paper is to present a TDR-based soil moisture monitoring system with simultaneous measurement of soil temperature and electrical conductivity developed at the Institute of Agrophysics PAS (Lublin, Poland), and implemented in the Polesie National Park in Poland. Specifically, the main objective is to describe the applied time delay units and the effect of a temperature compensation step on the measurement accuracy. Other hardware issues, like the applied probe or the battery power supply, important for the overall performance of the system, are also described as a secondary objective. Additionally, the paper discusses a sample field application of the presented system for the purpose of the long-term soil moisture monitoring in Polesie National Park.

The presented monitoring system is a continuation of previous developments from the Institute of Agrophysics PAS. The basic element was a soil water content measurement unit working in the time domain reflectometry technique with a needle pulse analyzing signal, developed in the late eighties by Malicki and Skierucha [24]. A narrow needle pulse signal generator with sufficiently sharp rise and fall times is relatively easy to produce [24,25], compared to a step pulse generator. Needle pulse reflections from the TDR probe are easier to interpret and analyze than those from a step pulse; the respective needle pulse generators and sampling heads can be galvanically isolated from the soil and the electronics of the measurement system works in a much narrow bandwidth compared to the step pulse technique. The majority of scientific work on the TDR technique for determining soil water content is based on interpreting step pulse reflections [11,26,27] produced by expensive equipment adapted from telecommunication (e.g., cable testers for locating cable faults in cable networks). The needle pulse TDR soil moisture content measurement systems equipped with electronic circuits available from the fast-growing high frequency mobile telecommunication industry can be priced competitively with the TDR soil moisture content measurement systems available on the market [12].

The apparent dielectric permittivity calculated from the velocity of propagation of the electric pulse in the soil is converted into the soil volumetric moisture content on the basis of the calibration given in [28]. The needle pulse TDR soil moisture meters were successfully used for implementing corrections due to soil density [28] and temperature [29,30] in the conversion functions from the soil dielectric permittivity to the soil moisture content. Also, soil salinity status [31] was assessed on the basis of the collected TDR-based measurements. Further work with a TDR needle pulse concentrated on the development of field monitoring systems of soil moisture, electrical conductivity and temperature [32]. After a TDR signal multiplexer [33] and hardware/software upgrade, which included wireless GPRS communication facilities and internet data management, the technologically updated monitoring system was constructed. The paper presents the following:

- the principle of simultaneous measurements of three physical quantities of soil implemented in the system, *i.e*., bulk dielectric permittivity *ε_b_*, bulk electrical conductivity *σ_b_* and temperature *T*,
- hardware elements of the system with special attention to: (i) the time delay units responsible for measurement accuracy and resolution of the soil dielectric permittivity and the results of the applied temperature compensation, (ii) integrated probe for simultaneous measurement of *ε_b_*, *σ_b_* and *T*, (iii) power consumption analysis of the battery operated monitoring system,
- effects of the applied temperature correction on the electronic time delay unit,
- measurement results collected over a two-year period from an implemented ground monitoring station.

## 2. Applied Measurement Principles of Soil Moisture Content, Electrical Conductivity and Temperature

### 2.1. Soil Moisture Content

The electromagnetic wave in a TDR parallel metallic probe of length *L* is assumed to propagate in the transverse electromagnetic mode, meaning that the electric and magnetic fields are transverse to the direction of the propagation of the wave. The propagation velocity *υ* of the TDR pulse in the soil is determined from [11]:

(1) $$\upsilon =\frac{1}{\sqrt{\left(\mu ɛ/2)(\sqrt{1+\tan^{2}\delta }+1\right)}}$$

(2) $$ɛ/{ɛ}_{0}={{ɛ}_{r}}^{\prime }-{j{ɛ}_{r}}^{\prime \prime }={{ɛ}_{r}}^{\prime }-j\frac{\sigma_{b}}{\omega {ɛ}_{0}}$$

(3) tanδ=Im(ɛ)Re(ɛ)=σbωɛr′ɛ0

where *ε* is the complex dielectric permittivity (with *ε_r_*′ and *ε_r_*″ as its relative real and imaginary parts, respectively, as compared to the values in free space), *σ_b_* (S/m) is the apparent or bulk electrical conductivity, tan*δ* is the loss tangent and *μ* is the magnetic permeability of the soil, *ω* (rad/s) is the angular frequency and *ε*_0_ = 8.85 × 10^−12^ (F/m) is the dielectric permittivity of free space.

For materials with a relatively low electrical conductivity and considering frequencies of the order of 100 MHz and higher, the wave propagation velocity along the parallel metal waveguide fully inserted into the tested material can be approximated by:

(4) $$\upsilon =\frac{2L}{t}≅\frac{1}{\sqrt{\mu \mu_{0}ɛ{ɛ}_{0}}}≅\frac{c}{\sqrt{{ɛ}_{b}}}$$

where $c=1/\sqrt{\mu_{0}{ɛ}_{0}}$ and *μ*_0_= 4π × 10^−7^ [V·s/(A·m)] are the velocity of light and magnetic permeability of free space, respectively, *t* is the time necessary for the pulse to cover the distance equal to 2*L*, which is the sum of the forward and reflected runs of the TDR pulse along the waveguide and ${ɛ}_{b}≅{ɛ}_{r}^{\prime }$ is the apparent or bulk dielectric permittivity of the soil. The relation between the propagation velocity of the electromagnetic pulse in the soil and its real part of the relative dielectric permittivity provides the basis of the measurement principle of the TDR technique.

When the soil around the rods of a TDR sensor is homogeneous and has electrical conductivity small enough for the reflected signal not to be attenuated below the detection level, which is true for most soils, the apparent dielectric permittivity is an averaged value of ${ɛ}_{r}^{\prime }$ in the vicinity of the sensor. Because of the unique polar structure of the molecules of water, its dielectric permittivity is many times greater than that of air, *ε_a_* = 1, and the solid phase of the soil, *ε_s_* = 3 – 5. Therefore, the bulk dielectric permittivity of soil, and other porous materials as well, depends on the volumetric water content of the tested object.

The TDR soil water content calibration function, which expresses the volumetric water content *θ_υ_* of a sample as a function of its bulk dielectric permittivity, *θ_υ_* = *f*(*ε_b_*), is universal for most mineral soils. One of the calibration functions presented in [11] has the form:

(5) $$\theta_{\upsilon }=-5.3\cdot 10^{-7}+2.92\cdot 10^{-2}{ɛ}_{b}-5.5\cdot 10^{-4}{ɛ}_{b}^{2}+4.3\cdot 10^{-6}{ɛ}_{b}^{3}$$

There are many other TDR soil moisture content calibrations based on empirical data that include soil texture [34], soil bulk density [28], temperature [30,35], or dielectric mixing models that treat soil as a mixture of four phases, *i.e.*, solids, air, free water and bound water [36–38]. The TDR technique enables determination of the soil *θ_υ_* with an accuracy of ±2% of the measured value in relation to the standard thermogravimetric measurement method [39,40], without the need for any soil-specific calibration.

### 2.2. Soil Electrical Conductivity

TDR devices may be also used to determine bulk electrical conductivity of the soil. The authors in [41–45] showed how the attenuation of the TDR trace can be used to calculate *σ_b_*. Following the thin-sample approach presented in [44], *σ_b_* can be described by:

6) $$\sigma_{b}=\frac{K_{p}f_{T}}{Z_{L}}$$

where *Z_L_* is the steady-state impedance (in ohm), *f_T_* is a temperature correction coefficient, and *K_p_* is the cell constant of the TDR integrated probe that can be determined by immersing the probe in solutions with known conductivity.

The relationship between the TDR measured bulk electrical conductivity of the soil and the soil solution electrical conductivity, *σ_w_*, which, in turn, can be related to the concentration of an ionic solvent, is more difficult to describe since it is highly dependent also on *θ_υ_* and soil texture. As for the *ε_b_*(*θ_υ_*) relationship, several types of models have been proposed for the relationship among *σ_b_*, *σ_w_* and *θ_υ_*, e.g., purely empirical models [45], empirical-conceptual models [46], and physical-conceptual models [47]. However, it is important to note that all of these models have serious drawbacks in that, for example, they need to be calibrated for each soil type and are only applicable for a specific range of *θ_υ_* and *σ_w_*, where the reflected TDR signal is not completely attenuated. Thus, there is no universal theory for the relationship among *σ_b_*, *σ_w_* and *θ_υ_*, making novel approaches appealing [48].

### 2.3. Soil Temperature

The measurement of soil temperature *T* provides necessary information for the temperature correction of bulk soil electrical conductivity (see Equation (6)) and soil moisture content [30,35]. Significant fluctuations of soil moisture data, which were obviously correlated with soil temperature, were noticed with the introduction of soil moisture field monitoring systems based on reflectometric meters. The experimental evidence showed that the observed temperature effect on the TDR determined bulk dielectric permittivity is the result of two competing phenomena; *ε_b_* increases with temperature following the release of bound water from soil solid particles and *ε_b_* decreases with temperature increase following the temperature effect of free water molecules. Soil type, especially soil specific surface that is positively correlated with the amount of soil bound water, can determine the dominant phenomenon.

## 3. Applied Hardware and Software

**Figure 1.**
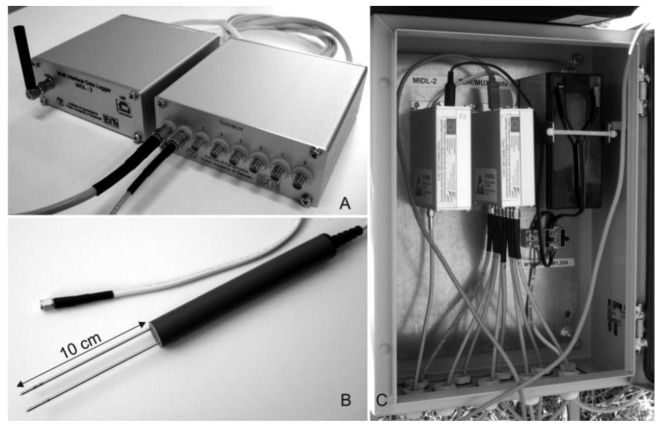
Elements of the telemetric monitoring system: (**A**) measurement unit type TDR/MUX with a GPRS modem [49], (**B**) integrated FP/mts probe for the measurement of soil moisture, temperature and electrical conductivity, (**C**) complete monitoring setup in a metal enclosure.

The presented soil moisture, temperature and electrical conductivity monitoring system includes the following elements: (i) eight-channel measurement units type TDR/MUX (Figure 1(A)), (ii) soil moisture, electrical conductivity and temperature two-rod probes, type FP/mts (short for **F**ield **P**robe for the measurement of soil **m**oisture, **t**emperature and **s**alinity) (Figure 1(B)), (iii) a GPRS modem controlled by an internet server for collecting data from the monitoring stations and data distribution among the users (Figure 1(A)—the device on the left).

In a sample field application discussed later in this paper, the FP/mts probes are located at the end of 6 m length coax 50 ohm feeder cable, type BELDEN 9907. They are installed in pairs at 10 cm and 50 cm below ground level. The data from the upper probe will be correlated with satellite data (Soil Moisture and Ocean Salinity (SMOS) Mission [23]), while the data from both probes, together with precipitation and evapotranspiration data from other sensors, will give information about water transport parameters in the soil at the selected sites. A detailed interpretation of collected data will be the subject of a future work.

The measurements of the three soil variables from each FP/mts probe: *θ_υ_*, *σ_b_* and *T* take place successively with 16, 8 and 1 repetitions, respectively. The mean values of data are stored in the internal data logger of the TDR/MUX device in the form of records with additional information: date, time, serial number of the meter and the measurement channel number. The data logger can upload these records by a GPRS link to the internet server for user access. Each user can login to their resources on the server using an internet browser to download the records and to modify the measurement time schedule. The monitoring station is supplied by a 7 VAh/12 V lead acid accumulator, which is charged by a solar panel.

### 3.1. TDR Soil Moisture Meter

**Figure 2.**
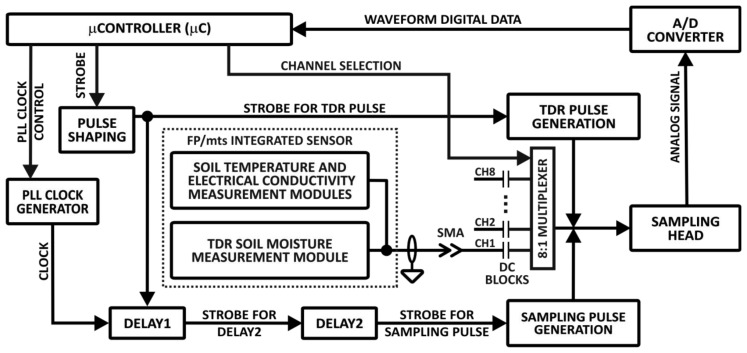
Functional elements of the TDR-based soil moisture monitoring system with simultaneous measurement of soil temperature and electrical conductivity.

**Figure 3.**
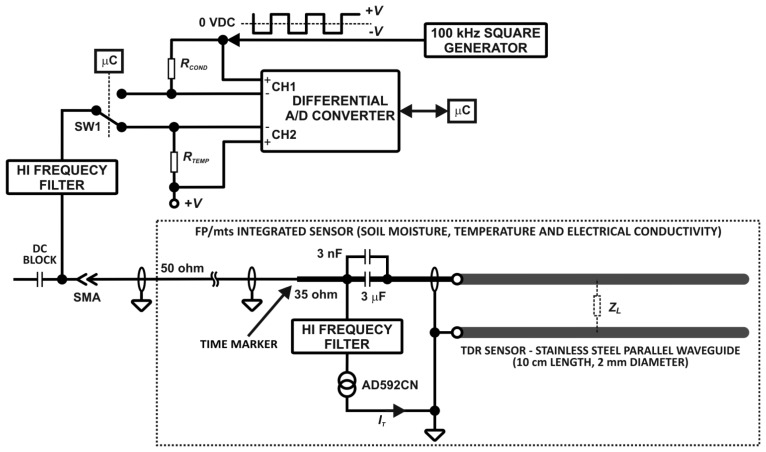
The functional details of the TDR integrated soil moisture, temperature and electrical conductivity probe with the respective signal and data processing hardware.

The functional elements of the TDR-based soil moisture monitoring system with simultaneous measurement of soil temperature and electrical conductivity are presented in Figure 2. The details of the TDR integrated soil moisture, temperature and electrical conductivity probe with the respective signal and data processing hardware are presented in Figure 3. The meter consists of several electronic modules controlled by a microcontroller (μC). The STROBE signal from the μC initializes sampling of a voltage value by the SAMPLING HEAD at a time determined by the two delay modules: DELAY1 and DELAY2. After forming the STROBE signal in the PULSE SHAPING circuit, the signal STROBE FOR TDR PULSE is generated. This is the input signal for two modules: (i) TDR PULSE GENERATION and (ii) DELAY1. The TDR PULSE GENERATION module produces a Gaussian needle pulse, with the rise and fall times of about 200 ps. This signal is fed to the input of a coaxial cable (SMA type coaxial connection), which connects the meter with a TDR integrated probe placed in the material under test [24,25,50].

The needle pulse travels through the coaxial cable of 50 ohm impedance (Figure 3), reflects from impedance discontinuities of the TDR integrated probe and returns to the SAMPLING HEAD, where the signal is sampled, integrated and converted into a digital form for further processing by the μC.

### 3.2. Integrated TDR Soil Moisture, Temperature and Electrical Conductivity Probe

**Figure 4.**
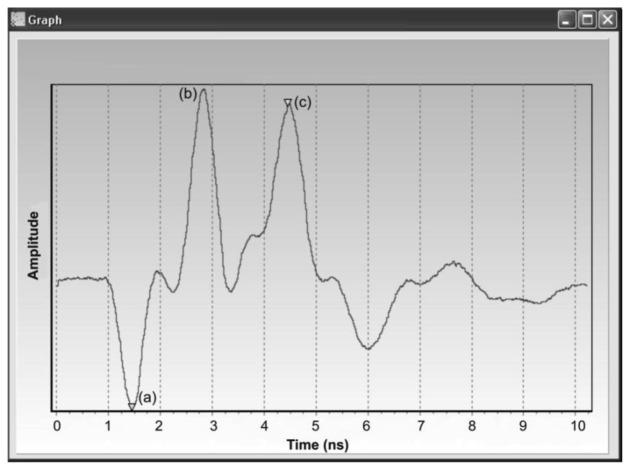
Screenshot of the output waveform generated by a computer application software, presenting a sampling window of 10 ns width with resolution of 10 ps: (a) reflection from the TIME MARKER, (b) reflection from the beginning of the metal rods, (c) reflection from the open termination of the metal rods.

The functional details of the TDR integrated soil moisture, temperature and electrical conductivity probe are presented in Figure 3. The probe consists of a strip-line of about 35 ohm and 7 cm length with characteristic impedance made from epoxy resin laminate. The connection between 50 ohm coax cable with the strip-line produces negative reflection (TIME MARKER, see Figure 4 point (a)). Two capacitors connected in parallel form a DC block for galvanic isolation forcing the direct current to pass the AD592CN temperature sensor [51]. The TDR pulse with high frequency components easily passes the 3 nF capacitor made of wide bandwidth dielectric material, reaches the rods of a parallel waveguide and is reflected from the successive impedance discontinuities, while the square waveform of 100 kHz frequency, for the measurement of soil electrical conductivity, passes the 3 μF capacitor without losing its symmetry. Maintaining the symmetry of this signal is necessary to avoid the effect of the sensor electrodes' polarization that can distort the measurement of the soil bulk electrical conductivity. The epoxy resin laminate with soldered coax cable, electronics and a short part (about 1 cm) of the parallel waveguide are inserted into a section of a PVC tube (13 cm length and 2 cm inner diameter) and filled with epoxy resin to form a mechanically stable construction for field use (Figure 1(B)). The implementation of the SW1 semiconductor switch characterized by low on resistance *R*_SW1_, allows changing the measured values between soil temperature and electrical conductivity, which are represented by voltage drops on the resistors *R_T_* and *R_COND_*, respectively, and measured by two channels of the differential analog to digital converter. The temperature dependence of *R*_SW1_(*T*) will also be accounted for in the temperature calibration of the TDR/MUX meter. High frequency filters, the DC block and the SW1 switch are used for selection of the measurement signals carrying information about water content (electric pulses with high frequency components), electrical conductivity and temperature of the soil.

In the presented monitoring system, the soil temperature is measured by an electronic temperature dependent current source [48], type AD592CN. The output of AD592CN has a highly linear characteristic with a slope 1 μA/K, excellent linearity below 0.15 °C in the temperature range from −25 °C to 105 °C and minimal self-heating errors.

Each TDR integrated probe is connected by an SMA coax connector to the TDR/MUX meter, which generates necessary signals and reads the respective responses. The current value *I_T_* depending on soil temperature value *T* corresponds to the voltage drop across the *R_TEMP_* resistor:

(7) $$I_{T}=\frac{V}{R_{\text{TEMP}}}+\Delta I(T_{\text{METER}})$$

and soil bulk electrical conductivity *σ_b_* corresponds to the voltage drop across the *R_COND_* resistor:

(8) $$\sigma_{b}=\frac{2VR_{\text{COND}}}{R_{\text{COND}}+Z_{L}}+\Delta \sigma_{b}(T_{\text{METER}})$$

where *R_TEMP_* and *R_COND_* are highly temperature stable resistors, Δ*I*(*T_METER_*) and Δ*σ_b_*(*T_METER_*) are appropriate corrections to compensate for the temperature drift of the electronics in the meter working in field conditions, *i.e.*, when the temperature of the meter *T_METER_* changes with the ambient temperature. The empirical determination of Δ*I*(*T_METER_*) gives the temperature measurement error of ±0.5 °C and Δ*σ_b_*(*T_METER_*) is less than 10% of the measured value. The temperature drift of the delay units will be described in the following section.

An example of a real reflectogram obtained from a TDR integrated probe placed in soil is presented in Figure 4. The first negative reflection (a) represents a TIME MARKER, introduced here to localize the measurement time window and eliminate the effect of the superposition of the signals reflected from the beginning and the end of the probe rods in the case of short propagation times, which occurs for dry soils [52]. The first positive reflection (b) comes from the beginning of the parallel waveguide rods and the second one (c) results from the reflection from the ends of the waveguide rods.

### 3.3. Delay Units

The temporal and temperature stability of the delay units in the TDR meter define the accuracy of the measurements of the time distances in the collected waveform and, consequently, the accuracy of the soil moisture content measurement. The delay unit of the TDR meter (Figure 2) consists of two modules: (i) DELAY1 for localizing in time the beginning of the sampling window for the signal reflected from the TDR integrated probe, and (ii) DELAY2 for triggering sampling pulses in the SAMPLING HEAD that collects a waveform reflected from the TDR integrated probe in the previously determined sampling window. A detailed description of these units is presented below.

#### 3.3.1. DELAY1—Cable Length Compensation

The first delay unit DELAY1 compensates for the cable length connecting the measuring device with the TDR integrated probe. A fixed and usually long cable of a TDR integrated probe and a fixed delay value of DELAY1 is not practical for the following reasons: (i) excessively long cable causes signal amplitude attenuation; (ii) temperature changes influence the cable unit delay, which in turn would cause a shift of the sampling window beyond the working range and (iii) any accidental shortening of a cable in harsh field use would make the TDR integrated probe permanently unusable. Furthermore, when a broadband multiplexer [33] is used for switching between TDR, each located several metres apart, there is a risk of damaging the TDR input circuits because of the difference in electric potentials between the probes. In this paper it is assumed that the cable length can be variable and the device automatically adjusts the delay value of DELAY1 through detecting the reflection from the TIME MARKER (Figure 3), which defines the beginning of the sampling window on the time scale. This marker is produced by an impedance discontinuity located a few centimetres before the parallel waveguide placed in the tested material. Assuming the length of the parallel rods of the TDR integrated probe *L* = 10 cm, the maximum width of the sampling window for a TDR integrated probe put in water with the relative dielectric permittivity *ε_w_* = 80 at room temperature is equal to:

(9) $$\text{DELAY}2_{\mathrm{MAX}}=\frac{2L\sqrt{{ɛ}_{w}}}{c}≅6\text{ns}$$

where *c* = 3 × 10^8^ m/s is the speed of light in vacuum.

**Figure 5.**
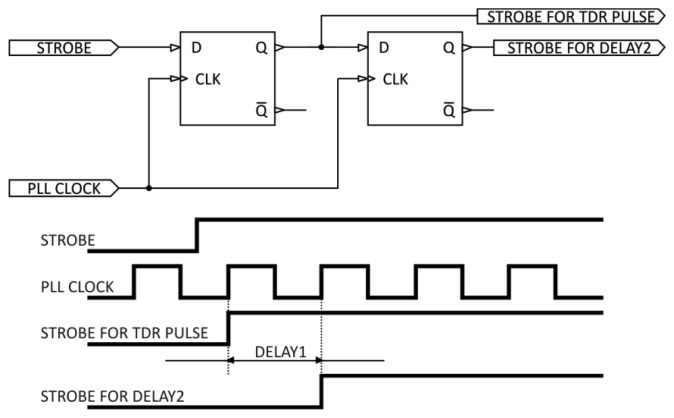
Principle of operation of DELAY1 unit for cable length compensation.

The delay time distance of the DELAY1 unit is generated by a programmable clock synthesizer working in a PLL integrated circuit, e.g., AD9552 from Analog Devices [53]. The principle of operation of this module is presented in Figure 5.

The clock signal PLL CLOCK, whose frequency may be programmed by entering the appropriate control values into the control registers of the synthesizer unit, is fed to the CLK input. The operation of the unit is initiated by the trigger signal (STROBE) from μC to the D input of a D-type flip-flop. The rising edge of the CLK latches the value of the STOBE line, changes the Q output of the first flip-flop and generates the STROBE FOR TDR PULSE signal, which goes to the TDR PULSE GENERATION module. The Q output goes to D input of the second flip-flop. The rising edge of the PLL CLOCK latches the “1” value on the output of the second flip-flop and changes its Q output, generating the STROBE FOR DELAY2 signal.

The difference in time between the STROBE FOR DELAY2 and STROBE FOR TDR PULSE signals compensates for the propagation time of the pulse in the coax cable (feeder), which transmits the pulse to the parallel waveguide of the TDR integrated probe. With the TDR sensor placed at the end of the coax cable of an unknown length, the control module moves the sampling window in time, searching for the TDR integrated probe marker. The algorithm of the automatic compensation of the cable length is as follows: the control unit sets the clock frequency to 400 MHz, generating a time interval DELAY1 = 2.5 ns, which represents the cable length *L* ≈ 0.5 m. The sampling window is therefore set to sampling of the time interval from 2.5 ns to 12.5 ns from the STROBE signal. If no TIME MARKER is found in this interval, the control unit sets the clock frequency to 80 MHz, generating a time interval DELAY1 = 12.5 ns, representing the cable length *L* ≈ 2.47 m. Then the sampling window encompasses the time interval from 12.5 ns to 22.5 ns from the STROBE signal. After subsequent shifts of the sampling window, the TIME MARKER is found and the control unit adjusts the generator frequency so that the sampling window includes the TIME MARKER and the whole reflectogram from the TDR integrated probe (Figure 4).

The discretization of the clock frequency selection of the PLL CLOCK is the result of the operation principle of the phase-locked loop based clock generators with integrated VCO – Voltage Controlled Oscillator [54]. Because of that, the accuracy of the cable length to the marker estimation is lower for longer cables. For the applied PLL based clock generator, the clock frequency change of 1 MHz causes a much larger increase in the cable length compensation time of DELAY1 for longer cables (lower frequencies generated) than for shorter ones (higher frequencies generated). For a coaxial cable of the BELDEN 9907 type, the sum of forward and return propagation times is about 5 ns/m and for the cable length of 8 m the 1 MHz frequency change is equivalent to about 0.3 m cable length.

Continuous technological progress in electronics, especially in integrated circuits in the field of high frequency communication devices, allows choosing the most suitable module for the defined application. For example, the AD9552 chip from Analog Devices gives a very flexible and precise frequency selection of the internal VCO oscillator up to 4 GHz, an output frequency up to 800 MHz and RMS jitter (small rapid variations in a waveform timing) less than 0.5 ps. As a result, the sensitivity of the DELAY1 module to frequency change of the PLL clock generator can be minimized to achieve practically full time variability of the DELAY1 unit and the corresponding compensation of the cable length.

It should be stressed that designing electronic devices working in high frequency range involves the application of specific techniques for impedance matching, supply filtering and implementation of fast digital circuits working in ECL or PECL techniques.

#### 3.3.2. DELAY2 in Equivalent-Time Sampling

**Figure 6.**
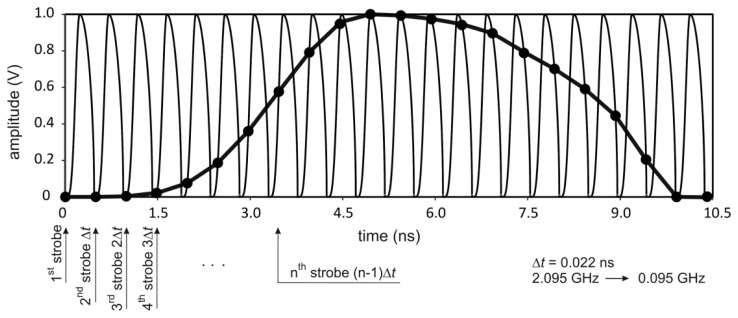
The principle of the equivalent-time sampling used in the TDR soil moisture meter.

The TDR technique uses high frequencies (of the order of 1 GHz) and requires measurements of relatively short time intervals between reflections of the pulses in a TDR integrated probe (with time resolution in the range of 10 ps). Because of that, it is not possible to use standard real-time measurement techniques. Therefore, the stroboscope or equivalent-time sampling method is applied [55], the principle of which is presented in Figure 6. This technique allows for conversion of high operation frequencies of the device to much lower frequencies. This in turn enables further signal processing to be performed by much cheaper and energy-saving standard electronic systems.

In the equivalent-time sampling, a repetitive train of identical pulses is applied to the input port; the sampling circuit is used to reconstruct the shape of an individual pulse from the input pulse train. This is accomplished by firing the strobe during each repetition of the input pulse train at a time Δ*t* later than it fired in the previous cycle of the input pulse train. In this way the strobe firing time slowly “scans” across the input pulse that is being sampled. Since each successive digitized voltage sample corresponds to an input voltage at a short time Δ*t* later than the previous voltage sample, the shape of the pulses in the input pulse train can be reconstructed from the digitized output voltage record. The equivalent-time sampling from Figure 6 produces the frequency conversion from 2.095 GHz to 0.095 GHz with the time shift Δ*t* = 0.022 ns.

Generation of the STROBEFORSAMPLINGPULSE signals can be done through the application of programmable delay integrated circuits working in various techniques. The AD9501 unit from Analog Devices [56] uses a ramp/comparator/DAC architecture [57]. One input of a high speed comparator is driven by a digital-to-analog converter (DAC). The DAC is used to set a reference voltage at this comparator input. The other input is connected to a ramp generator, which is started by applying a pulse to the trigger input of the delay generator. When the ramp voltage crosses the comparator threshold set by the DAC, the output of the comparator switches. The full scale range delay can vary from 2.5 ns to 10 μs and beyond and is programmed by external RC passive elements. The minimum time shift Δ*t* is 10 ps and only 256 steps of the delay are possible, which is a great disadvantage for high time-resolution systems.

**Figure 7.**
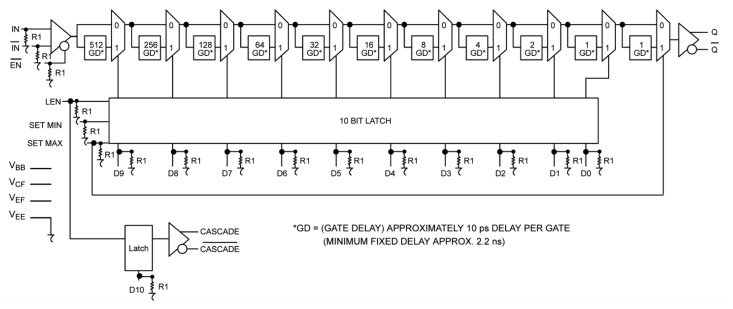
Logic diagram of the MC100EP195 programmable delay chip from ON Semiconductor [58].

The DELAY2 unit applied in the presented solution uses an integrated circuit MC100EP195FA from ON Semiconductor with a selectable delay from 2.4 to 12.4 ns with 10 ps time increments. The operating principle of a delay line is based on commutating its active elements with times proportional to a binary code with a step of 10 ps (10, 20, 40 ps, …). The delay unit contains a programmable gate array and a multiplexer (Figure 7). The required delay time is set in ten input data lines D9–D0 with the aid of the control signal LEN. The chip has a fixed initial delay time of 2.4 ns, because it incorporates an internal multiplexer. There is a possibility of cascading several chips. All components of the MC100 family have temperature compensation, but according to the catalogue data this compensation is not strong enough to account for the proper work of the meter in field conditions.

For example, the temperature change of the MC100EP195 from 25 °C to 85 °C can increase the programmable delay up to Δ*t* = 1,000 ps, which for the 10 cm length of TDR integrated probe rods can increase the absolute measurement error of TDR determined volumetric moisture content Δ*θ_υ_* ≈ 10% [40]. Therefore, the temperature calibration of the MC100EP195 and its linearity should be checked experimentally for each item of the TDR/MUX meter to reduce the Δ*θ_υ_* below the value that results from the variability of soil texture and density. The maximum absolute error of time distance measurement was assumed to be Δ*t_max_* = 30 ps, which corresponds to the value of Δ*θ_υ_* ±0.3% [40]. In the presented measurement system, the MC100EP195 electronic chip in the TDR/MUX meter is equipped with a temperature sensor of type PT1000, which is attached by thermo-conductive glue to the top of its ceramic enclosure. This temperature represents the temperature of the TDR/MUX meter and it is monitored and further processed by means of the central μC for implementation of the necessary temperature corrections described below.

Each of the applied TDR integrated probes connected to the TDR/MUX units measures three parameters: soil volumetric water content, temperature and electrical conductivity, simultaneously and from the same sample volume. Calibration of the probes and the discussion of errors generated by the diversity of the measured material are presented in other papers [30,40,52] and the discussion below concentrates on the temperature influence on the DELAY2 unit of the TDR/MUX meter. Due to the complexity of electronics in the applied hardware and no detailed catalogue information about the temperature drift of time delay integrated circuits similar to MC100EP195, each of the TDR/MUX meters was individually subjected to the temperature *T* change from −10 °C to 50 °C in a temperature chamber to identify the necessary compensation of the temperature drift introduced by the measurement hardware. Each measurement channel of the TDR unit was connected to a calibration box located outside the temperature chamber in a stabilized room temperature of 20 °C ± 1 °C. The calibration box consisted of 10 sections of different length of a RG316 coax cable, each one simulating various soil apparent dielectric permittivity *ε_b_*. The whole system, *i.e.*, the temperature chamber, the calibration box and the TDR/MUX unit, was controlled by a software application from a PC compatible computer. After the temperature *T* equalized with the temperature of the measurement unit (determined by the PT1000 temperature sensor mentioned earlier), the system automatically measured the “soil artificial” value of the bulk electrical permittivity *ε_b_*(*T*) from the propagation velocity of the pulse along the RG316 coax cable sections of various lengths using Equation (4). The temperature correction of the TDR/MUX meter was done for only one channel as the frequency and time domain performance of each channel was the same. The value of the time delay *t_T_* produced by the DELAY2 unit at *T* temperature is described as:

(10) $$t_{T}=t_{20}+\frac{dt_{T}}{dT}(T-20)$$

where *t*_20_ is the time delay value in reference temperature of 20 °C.

### 3.4. Battery Supply Issues

**Figure 8.**
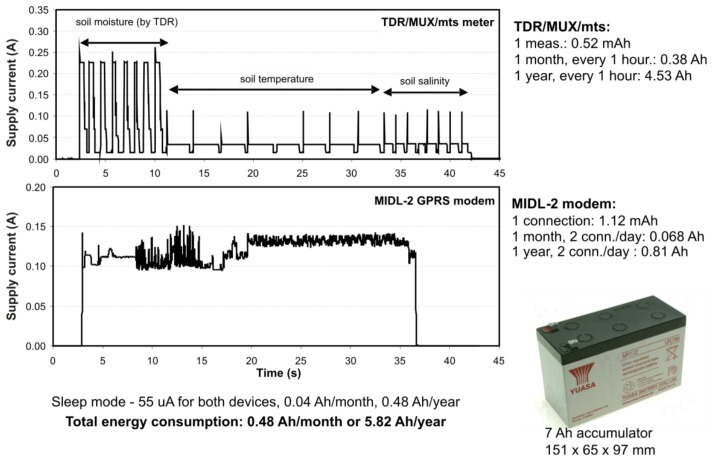
Energy balance of a single monitoring station of TDR soil moisture, temperature and electrical conductivity.

An important issue related to the performance of remote sensing devices is the energy budget. The presented monitoring system is powered from a lead acid accumulator with a capacity of 7 Ah. The upper part of Figure 8 presents the energy requirements of the monitoring station during one measurement cycle, which includes the measurements of three variables: soil moisture, temperature and electrical conductivity from one TDR integrated probe.

The lower part of Figure 8 presents the energy requirement of the GPRS communication unit for a single GPRS connection with the internet server. Each measurement series from eight probes, repeated every hour, consists in the measurement of eight values of soil moisture, temperature and electrical conductivity. The most energy from the supply battery is used in the process of the soil moisture measurement, which lasts about one second for each TDR integrated probe. Each series consumes 0.52 mAh of energy, 0.38 Ah monthly and in total 4.53 Ah for a year.

The GPRS connection by the MIDL-2 modem consumes 1.12 mAh and assuming two connections per day, gives a total of 0.81 Ah for a year. Such a calculation was necessary to select an accumulator of sufficient capacity to work for a period of at least one year. The practice confirmed the assumptions and calculations.

## 4. Results of the Applied Temperature Correction of the Time Delay Unit

**Figure 9.**
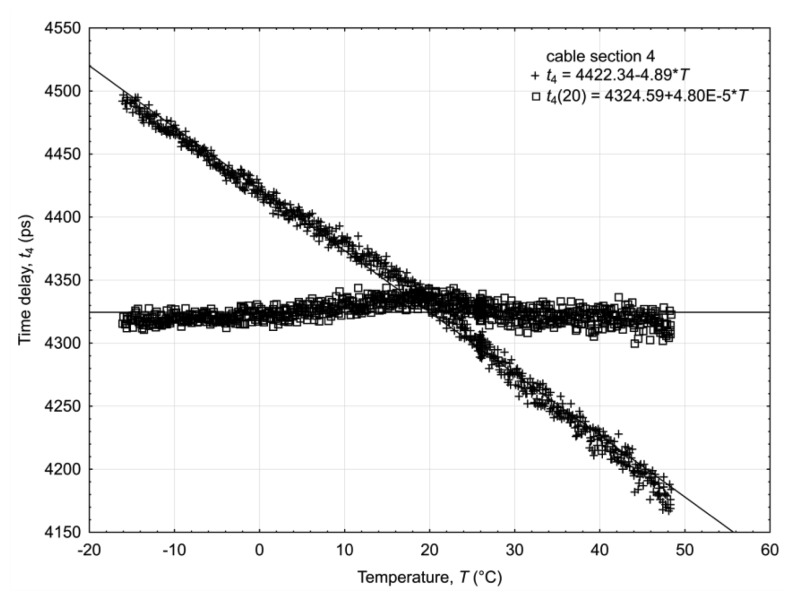
Example of the uncorrected (crosses) and corrected (squares) values of temperature relation of the example cable section No. 4 of the temperature correction setup.

The temperature time delay *t_T_* correction for the DELAY2 unit to the respective values at 20 °C is produced after transformation of the Equation (10). The *t*_20_ corrected value is:

(11) $$t_{20}=t_{T}-\frac{dt_{T}}{dT}(T-20)=t_{T}\left(1-\frac{dt_{T}}{dT\cdot t_{T}}(T-20)\right)$$

where $\frac{dt_{T}}{dT\cdot t_{T}}$ represents the unit temperature drift of the DELAY2 unit. The effect of the temperature correction of the DELAY2 unit affecting cable section No. 4 and described by Equation (11) is presented in Figure 9. After correction, the average value of the delay is 4,338 ps, with a standard deviation equal to 6 ps. The temperature-uncorrected and individually temperature-corrected values of delays introduced by the different lengths of coaxial cable sections, together with the corresponding statistics are presented in Table 1. The relative error (standard deviation divided by the delay value) decreased about ten-fold after correction (11), which is evident from Figure 9, where the uncorrected values are linear with the temperature with a much bigger slope than the corrected values.

The unit temperature drift $\frac{dt_{T}}{dT\cdot t_{T}}$ of the DELAY2 module was introduced to make the correction procedure convenient and easy to implement in the internal software of the microcontroller. The mean value for all channels in a TDR/MUX meter is −1.17 × 10^−3^ (1/°C) with a standard deviation of 4.2 × 10^−3^. The use of this value in the Equation (11) gives a slightly worse temperature correction compared to the individual correction, but still the relative error introduced by the temperature change of the DELAY2 unit decreased about eight-fold.

## 5. Example of a Field Application of the Presented Monitoring System and First Measurement Results

**Figure 10.**
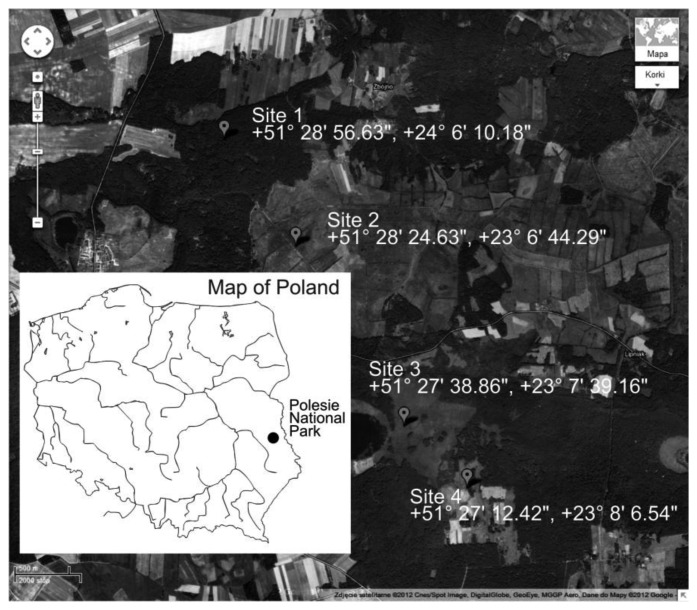
Selection of monitoring sites for soil moisture, temperature and electrical conductivity in Polesie National Park (source: Google Maps).

The main objective of the paper was to present the technical details of the TDR/MUX measurement devices and the following description of the obtained results and discussion should be regarded as exemplary. The measurement sites presented in Figure 10 were chosen for the purpose of long term monitoring of soil physical properties for comparison and correlation with atmospheric, geological and biotic characteristics of the monitoring sites.

**Figure 11.**
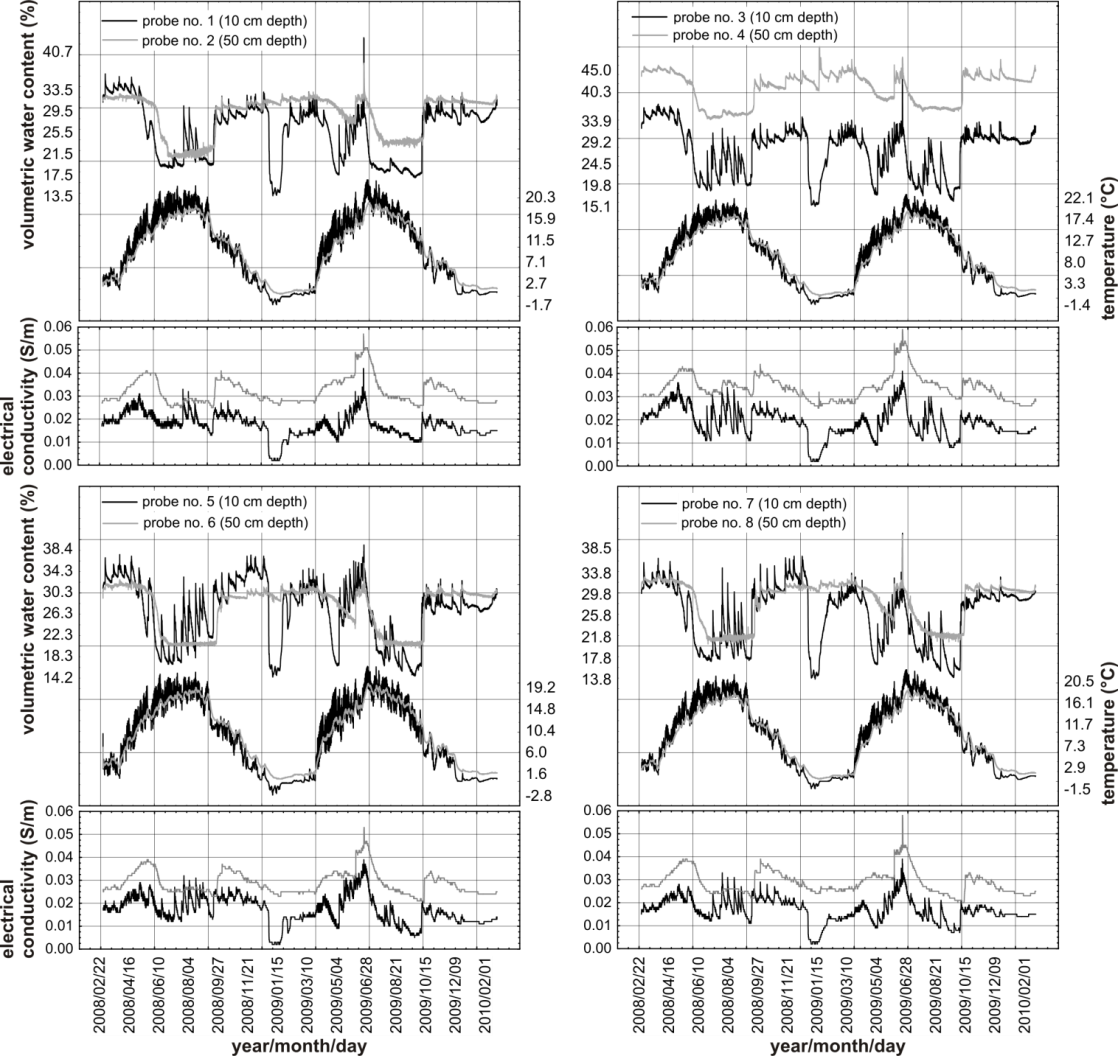
Temporal variability of soil moisture, temperature and electrical conductivity in Site 4 in Polesie National Park during the period 03.2008–02.2010.

Figure 11 presents the time variability of the values of moisture and temperature of the soil in the Site 4 measurement point (rendzina soil) in the Polesie National Park in eastern Poland. The Park covers an area of 97.64 km^2^, consisting mainly of forests, swamps, lakes and peat-bog terrains with numerous unique flora and fauna species.

The TDR integrated probes of the monitoring system described in Section 3 were placed horizontally at depths of 0.1 m and 0.5 m below ground in the walls of a circular hole with a diameter of about 0.5 m. The probes were calibrated with water and air as calibration media directly before their installation. As can be seen in Figure 11, despite the small distance between the probes, the values of volumetric water content and temperature at the same depth differ for the different probes. The variability of both measured quantities is greater at the depth of 0.1 m, where the influence of the precipitation and the ambient temperature is greater than at the depth of 0.5 m. The soil temperature only sporadically fell below 0 °C, despite severe frosts, especially during the winter of 2008/2009. The rapid decrease in measured soil moisture during winter was caused by a partial freezing of the soil water.

The relative dielectric permittivity of ice does not exceed the value of 5, while for the liquid water at a temperature near 0°C it equals about 90, which is why such measurement values were obtained by the TDR soil moisture meter. One may also notice the delay of the soil temperature and moisture changes at the depth of 0.5 m relative to the corresponding values at a depth of 0.1 m. The high correlation between electrical conductivity and moisture content confirms that soil electrical conductivity depends mainly on ionic electrical carriers that increase in number with soil moisture content [43,47]. Simultaneous measurement of soil electrical conductivity and bulk dielectric permittivity in the same soil volume can be used to determine soil salinity, *i.e.*, electrical conductivity of soil water extract [31,59]. Also, temperature dependent soil electrical conductivity can be corrected to the normalized value at 20 °C or 25 °C because both variables are recorded by the monitoring system.

The values of the physical quantities (soil moisture, temperature and salinity) measured by a TDR meter require correlation with corresponding atmospheric quantities if the TDR measurement results are to be used for modelling and forecasting purposes. The measurement data collected by the described system is uploaded to and distributed by the International Soil Moisture Network (ISMN). The ISMN system enables supplementing the soil moisture data at given locations with other physical parameters (metadata).

## 6. Summary

The discussed soil moisture content, temperature and salinity monitoring system represents current development trends in modern measurement systems featuring implementation of hardware and software procedures for ensuring high measurement accuracy, low power consumption and the possibility to control the measurement process from any place in the World using an internet or radio link in cases when access to the monitoring object is limited. High accuracy of the time delay units, described in detail, requires the use of sophisticated signal conversion integrated circuits and the application of temperature correction procedures. Exemplary results of the data collected from a ground monitoring station located in Polesie National Park have been presented. Analysis of soil moisture shows lower variability at greater soil depth and a correlation between soil moisture and soil electrical conductivity. The simultaneously collected temperature values in the same volumes as soil moisture content and soil electrical conductivity can be used for temperature correction of these variables. The system proved to be fully functional and economical, offering a cost-effective, reliable, and energy-efficient means for collecting distributed data. It is thus ready for commercialization stages.

## Figures and Tables

**Table 1.** Results of the temperature correction of the DELAY2 unit.

	
	**Cable section No.**									

	**1**	**2**	**3**	**4**	**5**	**6**	**7**	**8**	**9**	**10**
**Uncorrected average delay (ps)**	2,297	2,204	4,030	4,338	3,074	5,313	5,980	6,696	7,284	7,942
**Std deviation (ps)**	51	44	84	88	66	120	125	141	151	169
**Relative error**	2.23%	2.00%	2.09%	2.04%	2.15%	2.25%	2.10%	2.10%	2.08%	2.13%

**Average individually corrected delay to 20 °C (ps)—**Equation (11)	2,290	2,197	4,017	4,325	3,064	5,295	5,961	6,674	7,260	7,916
**Std deviation (ps)**	6	5	11	7	7	14	10	14	12	18
**Relative error**	0.27%	0.23%	0.27%	0.17%	0.24%	0.26%	0.17%	0.22%	0.17%	0.22%

**Unit temperature drift, $\frac{dt_{T}}{dT\cdot t_{T}}$ (1/°C)**	−1.23E−3	−1.11E−3	−1.15E−3	−1.13E−3	−1.19E−3	−1.24E−3	−1.16E−3	−1.16E−3	−1.15E−3	−1.18E−3

**Universally corrected delay to 20 °C using mean unity temperature drift (ps)**	2,289	2,196	4,015	4,322	3,062	5,293	5,958	6,671	7,257	7,913
**Std deviation (ps)**	7	6	12	10	8	17	12	17	15	21
**Relative error**	0.31%	0.27%	0.31%	0.22%	0.28%	0.33%	0.21%	0.26%	0.21%	0.26%
